# Chemical composition, antimicrobial, and antioxidant properties of essential oils from *Artemisia herba-alba* asso. and *Artemisia huguetii* caball. from Morocco: *in vitro* and *in silico* evaluation

**DOI:** 10.3389/fchem.2024.1456684

**Published:** 2024-12-09

**Authors:** Mohamed El Ouardi, Aziz Drioiche, Fadoua El Makhoukhi, Jamal Mabrouki, Mohammed Hakmi, Omkulthom Al kamaly, Bshra A. Alsfouk, Brahim Eddamsyry, Hamid Khamar, Touriya Zair, Mohammed Alaoui El Belghiti

**Affiliations:** ^1^ Research Team of Chemistry of Bioactive Molecules and the Environment, Laboratory of Innovative Materials and Biotechnology of Natural Resources, Faculty of Sciences, Moulay Ismaïl University, Meknes, Morocco; ^2^ Laboratory of Spectroscopy, Molecular Modelling, Materials, Nanomaterial, Water and Environment, CERNE2D, Mohammed V University in Rabat, Faculty of Science, Rabat, Morocco; ^3^ Higher Institute of Nursing Professions and Health Techniques of Fez, Regional Health Directorate Fez-Meknes, EL Ghassani Hospital, Fez, Morocco; ^4^ Medical Biotechnology Laboratory (MedBiotech), Bioinova Research Center, Rabat Medical and Pharmacy School, Mohammed V University in Rabat, Rabat, Morocco; ^5^ Department of Pharmaceutical Sciences, College of Pharmacy, Princess Nourah bint Abdulrahman University, Riyadh, Saudi Arabia; ^6^ Department of Botany and Plant Ecology, Scientific Institute, Mohamed V University in Rabat, Rabat, Morocco

**Keywords:** *Artemisia herba-alba* asso., *Artemisia huguetii* caball., camphor, thujone, eucalyptol, davanone, antimicrobial, antioxidant

## Abstract

**Introduction:**

Morocco is home to a remarkable diversity of flora, including several species from the Artemisia genus. This study aims to thoroughly examine the chemical composition of essential oils derived from Artemisia species and assess their antibacterial and antioxidant properties through in vitro experiments and in silico simulations.

**Methods:**

Samples of Artemisia herba-alba Asso. were collected from Boulemane and Ifrane in Morocco, while Artemisia huguetii Caball. was sampled from Tata, representing regions of the Central Middle Atlas and Western Anti-Atlas. Essential oils were extracted using hydrodistillation, and their chemical composition was analyzed by gas chromatography-mass spectrometry (GC-MS). Antibacterial and antifungal activities were evaluated, and antioxidant properties were assessed using the DPPH assay. In silico predictions of antibacterial and antioxidant activities were performed using computational models.

**Results:**

The extraction yields varied depending on the geographical origin, ranging from 1.54% to 2.78%. GC-MS analysis revealed significant differences in the chemical composition of the oils from different Artemisia species and regions, with a notable prevalence of oxygenated monoterpenes. Specifically, the oil from Boulemane was rich in thujone, the oil from Ifrane was predominantly composed of camphor, and the oil from Tata contained both camphor and thujone. The oils exhibited stronger antifungal than antibacterial properties, with Enterobacter cloacae strains showing high sensitivity, with minimum inhibitory concentrations (MIC) of approximately 12.5 mg/mL. The Boulemane oil of A. herba-alba displayed the highest antioxidant activity, effectively inhibiting DPPH at a concentration of 13.501 μg/mL.

**Discussion:**

The in silico simulations predicted that the primary compounds in these essential oils, such as davanone, eucalyptol, camphor, and thujone, would exhibit potent antibacterial and antioxidant properties. These compounds were found to have favorable ADMET characteristics, including good blood-brain barrier permeability, gastrointestinal absorption, and skin penetration. Molecular docking studies revealed strong interactions between these compounds and key target proteins, such as NADPH-dependent catalase and dihydrofolate reductase. The stability of the protein-ligand complexes was confirmed by molecular dynamics, with davanone showing a significant impact. Overall, this study provides a comprehensive understanding of the biological potential of Artemisia essential oils, highlighting davanone as a promising molecule for medicinal or pharmaceutical applications.

## 1 Introduction

In recent years, there has been a notable rise in research focused on exploring natural compounds and medicinal plants as potential substitutes for conventional antibiotics (Cook and Wright, 2022; Abdallah et al., 2023). This trend indicates increasing apprehensions over the worldwide epidemic of antimicrobial resistance (AMR), a growing public health issue exacerbated by excessive and inappropriate antibiotic use. The World Health Organization (WHO) has identified antimicrobial resistance (AMR) as a significant threat, leading to the creation of a Global Surveillance System to track and mitigate its fast proliferation globally (Talaat et al., 2022). Despite decades of research, gaps in current antibiotic development persist, with no new antibiotic classes introduced in over 50 years (Uddin et al., 2022), highlighting the urgency of finding alternative antimicrobial solutions.

Previous studies have investigated various approaches to leveraging plants for the treatment of bacterial and fungal infections (Salam et al., 2023). Essential oils derived from plants such as Artemisia exhibit potential owing to their extensive antibacterial capabilities. Recent literature demonstrates that essential oils possess significant antibacterial properties against resistant strains, offering a promising direction for research into new treatments (Barbieri et al., 2017; Ajeet, 2022). Components in essential oils, such as alkaloids, polyphenols, and terpenes, compromise bacterial cell membranes, resulting in cell death (Bouarab-Chibane et al., 2019; Ecevit et al., 2022).

The genus *Artemisia*, which belongs to the Asteraceae family and consists of around 500 species, is extensively distributed in the Southern Hemisphere as well as in North and East Africa (Nurlybekova et al., 2022). In Morocco, there are twelve species that represent *Artemisia*, including *A. herba-alba* Asso. and *A. huguetii* Caball. (Hinane et al., 2020). The *A. herba-alba* Asso, sometimes referred to as Chih in Arabic, is a perennial evergreen shrub that has the potential to grow between 15 and 60 cm in height. The flowering period of this plant starts in late summer, namely in September, and extends until December. The leaves of this plant are characterized by their woolly and hairy texture. *A. huguetii* Caball., often known as ifsi or izri, is found extensively in the region stretching from eastern Taznakht in the Ouarzazate province to the southern part of the Tata province. The *A. herba-alba* may be differentiated from the latter by its leaves that have long petioles and filiform lobes, small capitula that open when mature, and a spring phenology (Rafiq et al., 2016; Sanae et al., 2020).

Moreover, several ethnic groups have extensively employed the *A. herba-alba* species in traditional medicine to mitigate and cure diverse maladies, including diabetes, cancer, gastrointestinal and respiratory issues, as well as other disorders (Nigam et al., 2019; Bisht et al., 2021). Several pharmacological investigations have emphasized the significance of the subspecies *A. huguetii* Caball. and *A. herba-alba* Asso in traditional medicine. These species have shown a diverse array of biological and pharmacological capabilities, including antioxidant, insecticidal, antibacterial, antifungal, and anticancer activity, especially in relation to their essential oils (Bora and Sharma, 2011; Koul et al., 2017; Adewumi et al., 2020). Furthermore, the essential oils have demonstrated potential antioxidant properties by suppressing lipid peroxidation. These attributes might be advantageous in a range of biological systems and in the production of food (Ribeiro-Santos et al., 2018).

Recent research indicate that essential oils are potential agents against drug-resistant infections, especially multidrug-resistant bacteria and fungi. For instance, research by Somrani et al. (Somrani et al., 2020) highlights that specific essential oils can effectively penetrate bacterial biofilms, a known defense mechanism of resistant strains, thereby enhancing the efficacy of treatment. Additionally, studies by Nafis (Nafis et al., 2021) and Drioiche (Drioiche et al., 2024) illustrate how the synergistic action of essential oils with conventional antibiotics can significantly reduce the required dosage of synthetic antibiotics, thus potentially slowing the development of further resistance. These results highlight essential oils as both direct antimicrobial agents and as adjunct medicines that may enhance existing antimicrobial treatments, offering a dual strategy for controlling resistant infections.

Studies have been carried out on the antibacterial, antifungal, and antioxidant properties of essential oils derived from *A. herba-alba* Asso and *A. huguetii* Caball. However, it is important to note that these properties can vary depending on factors such as the extraction method, place of origin, and growth conditions. The chemical makeup of essential oils can significantly fluctuate depending on several conditions, which might directly influence their biological activity (Bertella et al., 2018; Ez zoubi et al., 2022; Polito et al., 2024).

This study aims to thoroughly investigate the chemical composition, antimicrobial, and antioxidant properties of the essential oils derived from *A. herba-alba* Asso. and *A. huguetii* Caball. plants in Morocco. The findings of this research have the potential to uncover new antibacterial compounds. The primary aim of this research is to enhance the comprehension of the volatile profile and the antibacterial, antifungal, and antioxidant properties of these particular essential oils, which are historically employed in Morocco and referred to as Chih and ifsi/izri. The *A. herba-alba* Asso. and *A. huguetii* Caball. subspecies exhibit variations in their timing of biological events, physical characteristics of their leaves, and geographical range within the taxonomic classification. In addition, the utilization of *in silico* techniques such as molecular docking, pharmacokinetics (ADMET), and drug similarity prediction will be preferred to enhance our comprehension of the mechanisms linked to these biological activities. The integration of computational methods with *in vitro* research will yield a more thorough and all-encompassing understanding of the characteristics and possible uses of these vital oils.

## 2 Materials and methods

### 2.1 Study area

The Central Middle Atlas of Morocco is characterized by a highly diversified bioclimate, including differences in climate, soil, and relief, as well as exceptional flora. This flora serves as a protective and productive heritage and also represents an important genetic reservoir for biodiversity. Additionally, the Western Anti-Atlas, a semi-arid to arid region with Atlantic and sub-Saharan influences, is known for its remarkable development of *Artemisia* steppe and its rich flora of aromatic and medicinal plants. This regional flora can be exploited to produce high-value products for the rural populations of the Boulemane, Ifrane, and Tata regions. The studied sites are located in the central mountainous area of the Middle Atlas and the oases of the Anti-Atlas.

**FIGURE 1:**
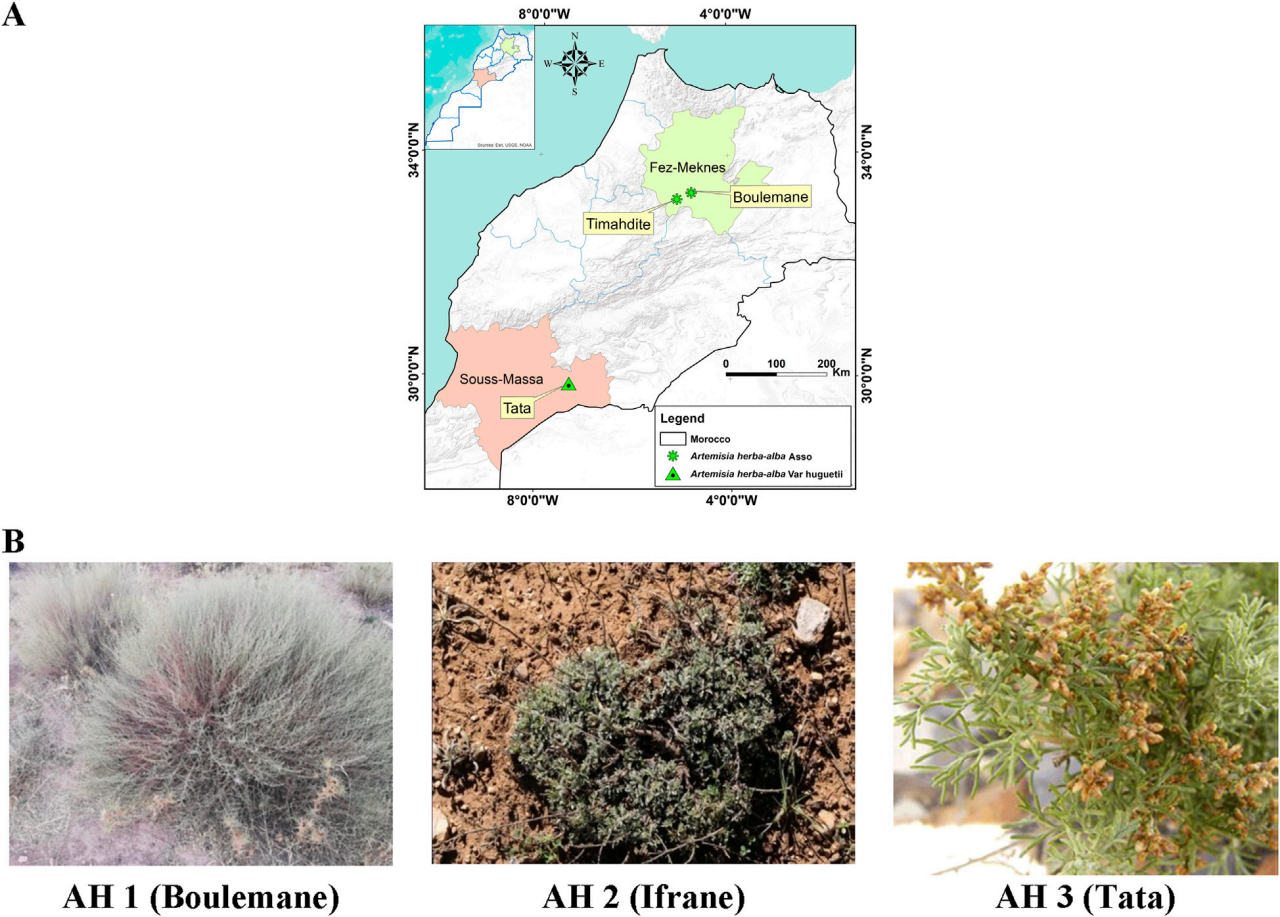
Collection sites for the studied *Artemisia* species **(A)**, along with the morphological characteristics of *A. herba-alba* Asso (AH1 and AH2) and *A. huguetii* Caball. (AH3) **(B)**.

The aerial parts (leaves and flowers) of three species of *Artemisia* (Asteraceae), namely *A. herba-alba* Asso (Boulemane) and *A. herba-alba* Asso (Ifrane), were gathered from two locations in the Middle Atlas: Boulemane and Ifrane. In the meantime, *A. huguetii* Caball. was collected in the Anti-Atlas, specifically in Tata, Tissint municipality (Figure 1A).

*A. herba-alba* Asso, a plant harvested from the Boulemane region, thrives in an average annual temperature of 17°C, with relatively low humidity around 40%, and receives moderate precipitation of about 300 mm per year. The soil in this region is characterized by a limestone base, clay-like texture, and a deficiency in significant organic material. Conversely, *A. herba-alba* Asso sourced from Ifrane grows in soil rich in organic matter, experiencing an average yearly temperature of 13°C, 60% humidity, and 600 mm of annual precipitation. In contrast, *A. huguetii* Caball. collected from Tata has an average annual temperature of 22°C, low humidity at 30%, and very limited precipitation averaging 100 mm annually. The plant grows in sandy, stony soil with poor organic content.

### 2.2 Plant material

The samples under investigation were taken in April 2022, during the flowering season. They were then allowed to dry for approximately 10 days in the shade. Prof. Hamid Khamar identified the species botanically at the Floristics Laboratory of the Scientific Institute of Rabat. Below (Figure 1B) are the morphological characteristics of *A. herba-alba* Asso (A and B) and *A. huguetii* Caball. (C).

### 2.3 Biological material

The bacterial and fungal strains were employed to evaluate the antibacterial activity of the essential oils of the studied *Artemisia* species. The bacterial strains included *Enterobacter cloacae*, *Klebsiella pneumoniae*, *Escherichia coli*, *Staphylococcus aureus* BLACT, and *Staphylococcus epidermidis*, while the fungal strains comprised *Candida albicans*, *Candida dubliniensis*, *Candida tropicalis*, *Candida parapsilosis*, and *Aspergillus niger*. These microorganisms, recognized for their robust resistance, invasive capacity, and toxicity towards humans, are unfortunately frequently encountered in numerous infections in Morocco, thus representing a genuine clinical and therapeutic challenge. These strains were isolated in a hospital setting, at the Mohamed V-Meknes Provincial Hospital, before being reconstituted in Mueller Hinton and Sabouraud broths containing 20% glycerol at −80°C, and this, prior to their utilization, after having been subcultured.

### 2.4 Essential oil extraction

The essential oils from the aboveground parts of the investigated *Artemisia* species were obtained by the hydrodistillation technique employing a Clevenger device. The procedure involved using 100 g of plant material, which was subjected to boiling in 1 L of distilled water for a duration of three hours. This process was repeated in triplicate to ensure accuracy. Subsequently, the essential oils were dehydrated using anhydrous sodium sulfate (Na_2_SO_4_) and stored in an opaque container at a temperature of 4°C until further use.

### 2.5 Analysis of essential oils using gas chromatography coupled with mass spectrometry

The chromatographic experiments were conducted using a Hewlett Packard gas chromatograph (HP 6890 series) equipped with an HP-5 capillary column (30 m × 0.25 mm × 0.25 µm film thickness) and a flame ionization detector (FID) that was calibrated at 250°C. The FID was maintained with a 30 mL/min hydrogen gas flow and 300 mL/min air flow to ensure high accuracy in compound detection. The setup was provided with a mixture of H₂ and air gases. Nitrogen was employed as the carrier gas in a split injection mode, with a flow rate of 1.7 mL/min. The column’s temperature was maintained at a constant rate of 4°C/min for five minutes, starting from 50°C and ending at 200°C. The HP ChemStation software, specifically version A.09.03, was used to oversee the device’s functions and monitor the chromatographic outcomes.

The GC-MS analysis was conducted using an HP 6890 chromatograph integrated with an HP 5973 series mass spectrometer, featuring electron ionization (EI) at 70 eV for fragmentation. The HP 5MS column with dimensions of 30 m × 0.25 mm × 0.25 µm was used. The column temperature was set at a steady rate of 4°C/min, starting from 50°C and reaching 200°C within five minutes. Helium, used as the carrier gas, was maintained at a flow rate of 1.7 mL/min, with split injection at a 20:1 ratio to enhance sample resolution. The chemical composition of the essential oil (EO) was determined by identifying and comparing the Kovats indices (KI) of the compounds with those of recognized standard products listed in the Kovats databases, namely Kovats (Kovats, 1965), Adams, R.P. (Adams, 2007), and Hübschmann, H.-J (Hübschmann, 2015). The compounds were identified by comparing their peak retention times with those of authentic known standards available in the authors’ laboratory, as well as by comparing their reported Kovats indices (KI) and mass spectrometry (MS) data with those stored in the WILEY and NIST 14 standard mass spectral databases (Version 2.2, 2014), as well as published literature. To ensure precision in identification, the Thermo Scientific TSQ 8000 Evo mass spectrometer (located in Waltham, Massachusetts, United States) was additionally used for verification, with comparisons made to the NIST/EPA/NIH Mass Spectral Library, Version 2.0, released in 2002.

### 2.6 Antioxidant activity of essential oils by DPPH radical test

The *In vitro* scavenging capability of the DPPH∙ free radical was measured using the procedure established by Nikhat (Nikhat et al., 2009). The experiment was conducted with a visible UV spectrophotometer set at a specific wavelength of 517 nm. The antioxidant capacity of different essential oils derived from three distinct species of *Artemisia* was assessed by employing DPPH∙ (1,1-diphenyl-2-picrylhydrazyl) as a rather stable radical. A solution of DPPH∙ was prepared by dissolving 2.4 mg of DPPH∙ in 100 mL of ethanol. To prepare the essential oils, dissolve them in a solution of two mg of ethanol per milliliter. The concentrations of this stock solution were acquired by successive dilutions, resulting in concentrations of 1, 2, 3, 4, 5, 6, 7, 8, 9, and 10 μg/mL. In order to carry out the experiment, 2 μL of the chemical being studied were mixed with 2.8 mL of DPPH∙ solution. For the purpose of positive controls, equivalent amounts of vitamin C, also known as ascorbic acid, were produced. The tests were conducted three times. Subsequently, the samples were placed in a lightless environment for a duration of 30 min, and the absorbances were quantified at a wavelength of 517 nm.

$$\%\text{ AA}=\frac{\text{Abs}\;\text{control}\;–\;\text{Abs}\;\text{sample}}{\text{Abs}\;\text{contr}ô\text{le}}\;x\;100$$

AA%: Percentage of antiradical activity.

Abs _control_: the absorbance measurement of the solution that contains solely the DPPH∙ radical.

Abs _sample_: absorbance of the sample solution to be tested in the presence of DPPH∙

The IC_50_, or concentration that corresponds to a 50% reduction in free radical activity, was found by graphing absorbance against essential oil concentration.

### 2.7 Determination of the minimum inhibitory concentration, the minimum bactericidal concentration, and the minimum fungicidal concentration

The BD Phoenix (Becton, Dickinson and Company, Franklin Lakes, NJ, United States) and VITEK2 (bioMérieux, Marcy-l'Etoile, France) automated systems were used to perform the antibiogram of the selected strains against the antibiotics (Gentamycin; Amoxicillin–Clavulanate; Vancomycin and Trimethoprim-Sulfamethoxazole). The clinical interpretation of antimicrobial susceptibility was conducted according to the current EUCAST guidelines (EUCAST, 2023a; EUCAST, 2023b). This antibiogram process on these automated systems involves evaluating bacterial growth in the presence of the antibiotic at different concentrations.

The minimal inhibitory concentration was established by the use of the microdilution method. A sterile 96-well microtitration plate was used to determine the minimum inhibitory concentration (MIC) of each essential oil (Balouiri et al., 2016). 100 μL of Sabouraud Dextrose Broth for fungus and Mueller-Hinton broth for bacteria were added to each well to create a microtitration plate. Concentrations ranging from 5 to 0.93 × 10^−2^ mg/mL for each essential oil were achieved by diluting a stock solution of essential oil made in 10% DMSO. Subsequently, a volume of 10 µL of inoculum containing 10^6^ CFU/mL of bacteria or 10^4^ CFU/mL of fungus was added to each well. The microplates were incubated at 37°C for a duration of 24–48 h. To assess microbial growth, 10 µL of resazurin solution (5 mg/mL) was introduced into each well following the incubation period. Microbial growth was indicated by a transition in hue from purple to pink after a second incubation period of two hours at 37°C. The MIC value is defined as the lowest concentration at which resazurin does not change color. To calculate the minimum fungicidal concentration (MFC) and minimum bactericidal concentration (MBC), 10 µL were extracted from every well that showed no apparent growth. These samples were then inoculated onto either Sabouraud agar for fungi or Mueller-Hinton agar for bacteria. The plates were incubated at 37°C for a whole day. The lowest concentrations of the examined samples that reduced 99.99% CFU/mL in comparison to the control were designated as the MBC and MFC. Antimicrobial potency is determined by calculating the MBC/MIC or MFC/MIC ratio. The essential oil has a bactericidal/fungicidal action if this ratio is less than 4, and a bacteriostatic/fungistatic effect if the ratio is larger than 4 (Bissel et al., 2014).

### 2.8 PASS, ADME, and the prediction of the toxicity analysis (Pro-Tox II)

The main compounds of the essential oils (Camphene, eucalyptol, cis-thujone, trans-thujone, yomogi alcohol, santolina alcohol, camphor, and davanone) of the *Artemisia* species studied were selected for PASS and ADMET prediction studies (Absorption, Distribution, Metabolism, Excretion, and Toxicity). To choose these compounds’ SMILES format, ChemBioDraw (PerkinElmer Informatics, Waltham, MA, United States, v13.0) (Li et al., 2004) was utilized. Subsequently, simulations were performed with the online prediction programs PASS-Way2Drug (Filimonov et al., 2014), SwissADME (Daina et al., 2017), and pkCSM for ADMET prediction. The terms “drug-like” chemicals’ potential activity (Pa) and likely inactivity (Pi) are referred to as PASS (Alam et al., 2021). We utilized the Pro-Tox II tool (https://tox-new.charite.de/protox_II/, accessed on 02 January 2024) to investigate toxicity levels and collect relevant information on important toxicological parameters such as LD50 and toxicity class (Banerjee et al., 2018).

The ADMET program, consisting of SwissADME, pkCSM, and Pro-Tox II, was employed to assess the chosen ligands. It was utilized to forecast the physicochemical attributes, lipophilicity, water solubility, pharmacokinetics, drug similarity, medicinal chemistry, and toxicological properties of the selected chemicals. Through the utilization of these methods and analytical tools, we obtained significant data on the potential therapeutic applications and potentially negative consequences linked to the primary chemical constituents found in the analyzed *Artemisia* essential oils.

### 2.9 Molecular docking protocol

Molecular docking is a critical computational method used to evaluate the binding capability of a ligand to the binding site of the target receptor. It considers alternative conformations and locations of the ligand (Moussaoui et al., 2021). For protein structure acquisition, the RCSB Protein Data Bank was accessed on 15 December 2023, and the structures were refined using PyMOL to enhance alignment with docking requirements. For antimicrobial activity, the target proteins included Glutathione S-transferase (PDB ID: 1a0f), Aspartic proteinase (PDB ID: 1j71), Beta-lactamase (PDB ID: 1xx2), Catalase compound II (PDB ID: 2cag), Sensor kinase CitA (PDB ID: 2j80), Endo-1,4-beta-xylanase I (PDB ID: 2qz2), Transcriptional regulator TcaR (PDB ID: 3kp2), Candidapepsin-2 (PDB ID: 3pvk), Kelch-like ECH-associated protein 1 (PDB ID: 6zez), Candidapepsin (PDB ID: 7agb), and Formate dehydrogenase (PDB ID: 8j3o). For antioxidant activity, the selected proteins included Superoxide dismutase (PDB ID: 1cb4), Dihydrofolate reductase (PDB ID: 2w9g), NADPH oxidase (PDB ID: 2cdu), Lipoxygenase-3 (PDB ID: 1n8q), Cytochrome P450 2C9 (PDB ID: 1og5), Xanthine dehydrogenase/oxidase (PDB ID: 3nrz), and Myeloperoxidase (PDB ID: 5qj2).

The ligands’ crystalline structures were acquired in sdf format from the PubChem database (www.pubchem.ncbi.nlm.nih.gov, accessed on 15 December 2023) and subsequently transformed into PDB format using the Open Babel software version 2.4.1 (O’Boyle et al., 2011). The ligands underwent optimization and energy reduction using the MMFF94 force field as specified by Kim and Lemkul (Kim et al., 2017; Lemkul, 2020). The receptor architectures were dehydrated and subsequently filled with polar hydrogen atoms and Kollman charges. The affinities of protein and ligand binding were determined by simulations using AutoDockTools-1.5.6, ensuring consistency in the generation of pdbqt files (Morris et al., 2009). The protein-ligand docking process was computed using AutoDock 4.2 with a grid box centered around the active site of each protein for optimal ligand alignment. The PyMOL 2.5.2 program was used to measure the root mean square deviation (RMSD) of heavy atoms between docked poses and crystallographic poses of ligands. To confirm docking accuracy, RMSD values under 2 Å were validated, as noted by Tabti et al. (Tabti et al., 2022). The Discover Studio 2021 visualizer (v21.1.0) was utilized to choose optimal postures based on docking scores. Furthermore, residue-ligand interactions were thoroughly examined and visually shown, as described by Meng (Meng et al., 2011).

## 3 Results

### 3.1 Extraction efficiency of the EO studied

The essential oil yields of the three studied *Artemisia* species exhibit variations as a function of their geographical provenance. The total yield percentages obtained were 1.54% ± 0.05% for the essential oil of *A. herba-alba* Asso originating from the Boulemane region, 2.09 ± 0,07% for that of *A. herba-alba* Asso from the Ifrane region, and 2.78 ± 0,10% for the essential oil of *A. huguetii* Caball. fom the Tata region. These results indicate that the *Artemisia* essential oil yield is influenced by the environmental conditions specific to each region of origin of the samples, a geographical variation that will need to be taken into account in the valorization and exploitation of these natural resources.

### 3.2 GC-MS analysis of the EOs of the *artemisia* species studied

**FIGURE 2:**
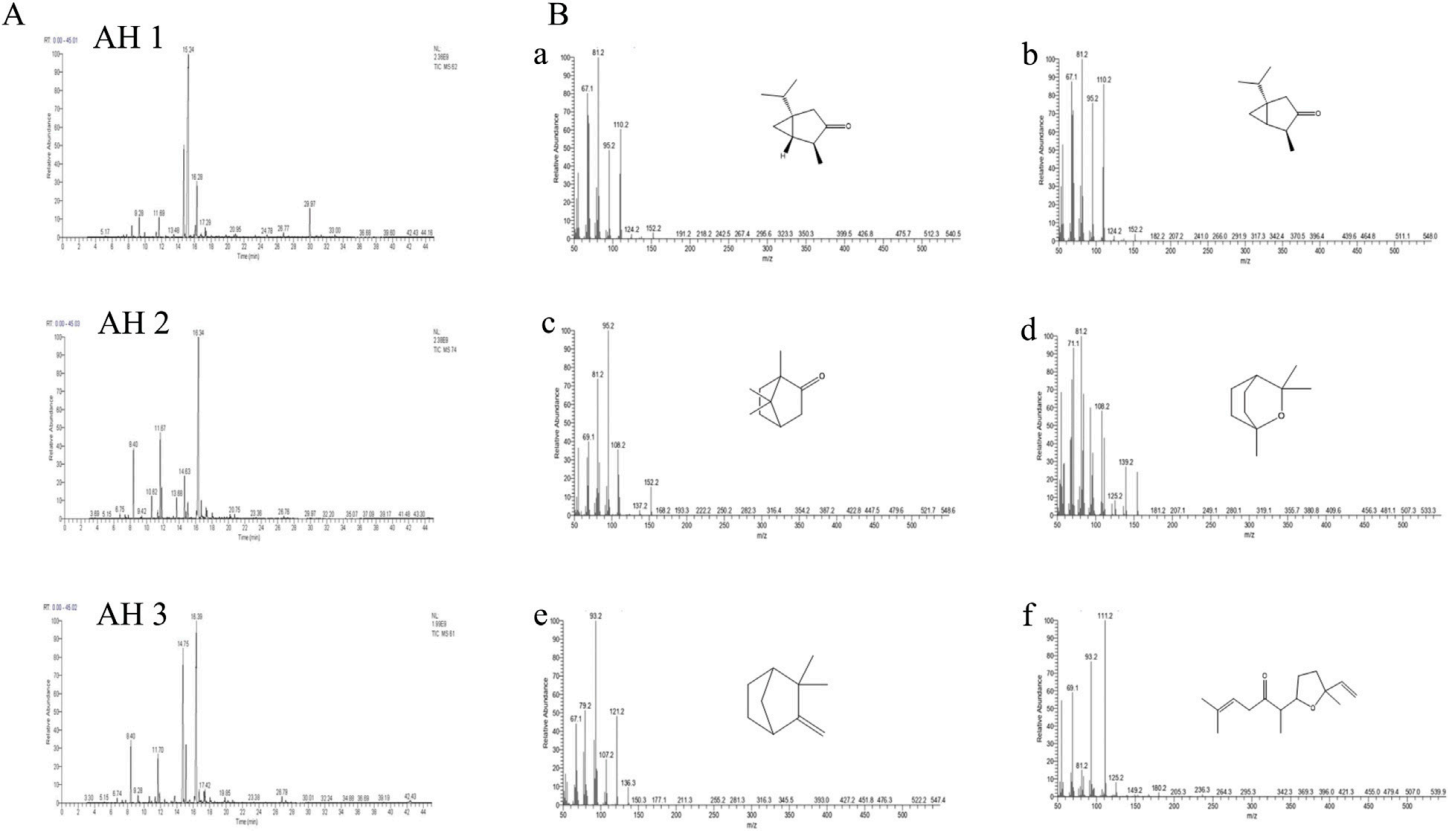
The chromatographic profiles of the *Artemisia* essential oils under study **(A)** and the mass spectra of the major compounds identified **(B)**: cis-thujone (a), trans-thujone (b), camphor (c), eucalyptol (d), camphene (e), and davanone (f).

The chromatographic profiles were measured following the results of chromatographic studies of the essential oils extracted from the studied *Artemisia* species to determine the different chemical compositions of the examined essential oils. There were some differences found between the three white *Artemisia* species as well as between the same species in various geographical locations (Figure 2A).

The chromatographic research of three *Artemisia* species essential oils showed that AH 1 oil from Boulemane and Ifrane oil both include 32 compounds, accounting for 99.79% and 99.58% respectively. Additionally, the study indicated that there are 31 components in total. Simultaneously, a total of 34 compounds, which make up 99.66% of Tata AH 3 EO, were discovered.

**FIGURE 3:**
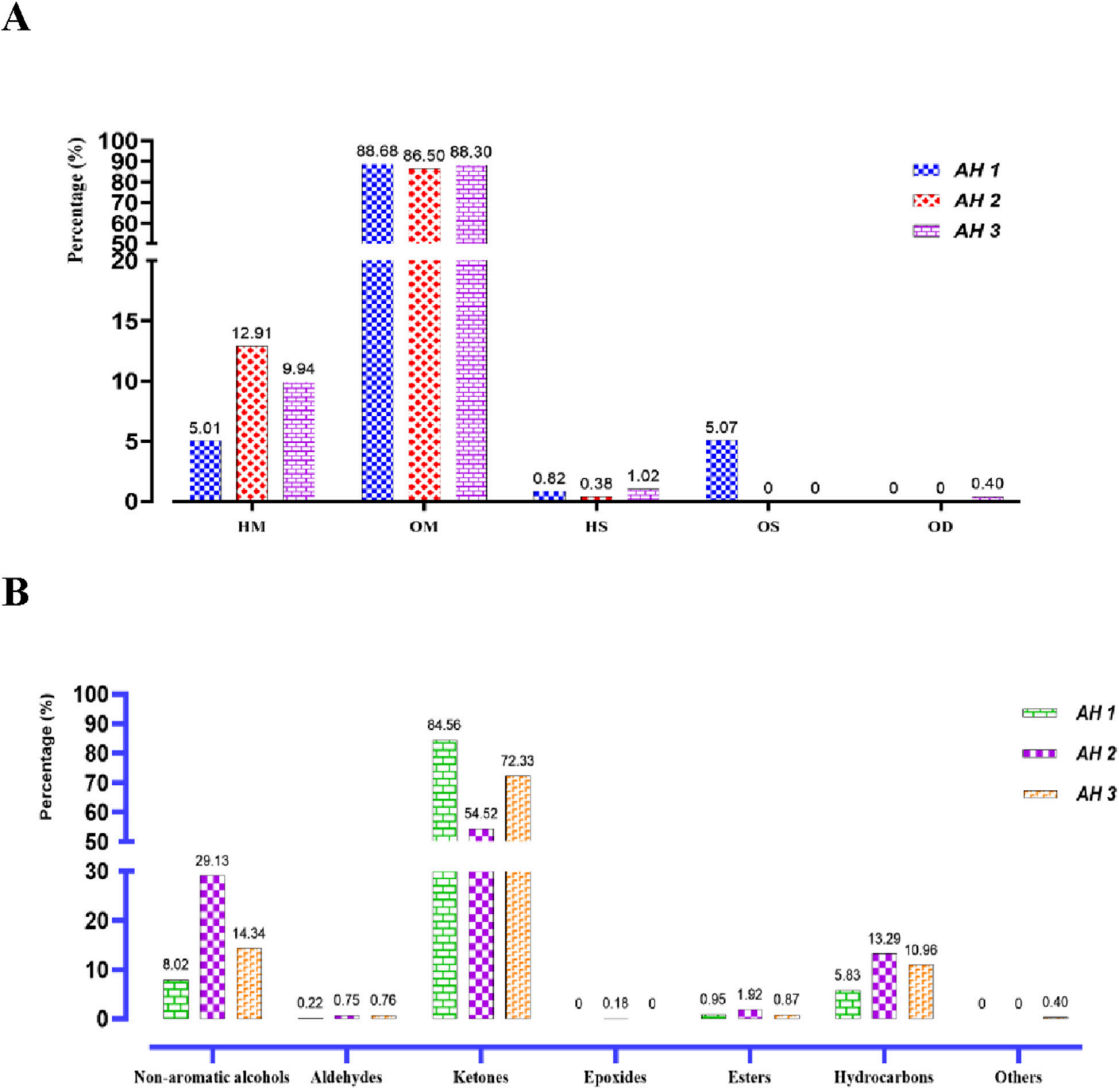
Distribution of the identified chemical classes **(A)** and families **(B)** in the essential oils of the studied *Artemisia* species. (HC, Hydrocarbon Monoterpenes; OM, Oxygenated monoterpenes; HC, Hydrocarbon sesquiterpenes; OS, Oxygenated Sesquiterpenes; OD, Oxygenated diterpenes).

The primary constituents of the essential oils from the three *Artemisia* species (AH1, AH2, and AH3) are mostly oxygenated monoterpenes, accounting for 88.68%, 86.50%, and 88.30% of the oils, respectively. In comparison, hydrocarbon monoterpenes, sesquiterpenes, and diterpenes are present in lower proportions in the three oils investigated (Figure 3A). The chemicals detected in the several species of *Artemisia* are present in variable proportions, as shown in Table 1.

**TABLE 1:** Chemical composition analyzed in the essential oil (EO) of *Artemisia* species.

Molecules	Chemical formula	Percentage of compounds			KI^a^
		AH 1	AH 2	AH 3	
Santolina triene	C_10_H_16_	0	0.58	0.48	908
2,5-diethenyl-2-methyl-Tetrahydrofuran	C_9_H_14_O	0.27	0	0	916
Tricyclene	C_10_H_16_	0	0.56	0.3	926
α-pinene	C_10_H_16_	0.3	0.42	0.28	939
Camphene	C_10_H_16_	1.26	9.07	6.71	954
Sabinene	C_10_H_16_	2.11	0.23	0.77	975
β-pinene	C_10_H_16_	0	0.38	0.19	979
Myrcene	C_10_H_16_	0.5	0	0	990
Yomogi alcohol	C_10_H_18_O	0	3.08	0.75	999
ρ-mentha-1 (7),8-diene	C_10_H_16_	0	0.32	0	1,004
α-terpinene	C_10_H_16_	0	0	0.17	1,017
ο-cymene	C_10_H_14_	0.65	1.11	0.68	1,026
Eucalyptol	C_10_H_18_O	2.29	11.68	5.23	1,031
Santolina alcoho	C_10_H_18_O	0	4.08	1.13	1,040
γ-Terpinene	C_10_H_16_	0.19	0.24	0.36	1,059
Cis-Sabinene hydrate	C_10_H_18_O	0.5	0	0	1,070
Artemisia alcohol	C_10_H_18_O	0	2.92	1.05	1,083
cis-thujone	C_10_H_16_O	15.38	8.96	27.38	1,102
Hotrienol	C_10_H_16_O	0	0.36	0	1,108
1,5,7-Octatrien-3-ol, 3,7-dimethyl-	C_10_H_16_O	0	0	0.26	1,108
trans-thujone	C_10_H_16_O	56.73	0	6.55	1,114
dehydro-sabina ketone	C_9_H_12_O	0.19	0	0.59	1,120
trans-ρ-mentha-2,8-dien-1-ol	C_10_H_16_O	0	2.43	0	1,122
Chrysanthenone	C_10_H_14_O	0.27	0	0	1,127
cis-ρ-mentha-2,8-dien-1-ol	C_10_H_16_O	0	0	1.42	1,137
iso-3-thujanol	C_10_H_18_O	1.87	0	0	1,138
trans-pinocarveol	C_10_H_16_O	0	0.95	0.6	1,139
cis-verbenol	C_10_H_16_O	0.76	0	0	1,141
Camphor	C_10_H_16_O	6.75	45.29	37.57	1,146
β-pinene oxide	C_10_H_16_O	0	0.18	0	1,159
Sabina ketone	C_9_H_14_O	0	0	0.24	1,159
Pinocarvone	C_10_H_14_O	0.4	0.27	0	1,164
cis-chrysanthenol	C_10_H_16_O	0	0.17	0.17	1,164
3-thujanol	C_10_H_18_O	0	0.22	0	1,168
Borneol	C_10_H_18_O	1.24	1.61	1.46	1,169
Terpinen-4-ol	C_10_H_18_O	0.68	1	1.44	1,177
2-Mmethyl isoborneol	C_11_H_20_O	0	0.24	0	1,181
Myrtenal	C_10_H_14_O	0.22	0.75	0.56	1,195
Myrtenol	C_10_H_16_O	0	0.39	0.35	1,195
trans-Ppiperitol	C_10_H_18_O	0	0	0.16	1,208
Cumin aldehyde	C_10_H_12_O	0	0	0.2	1,241
cis-chrysanthenyl acetate	C_12_H_18_O_2_	0.28	0.51	0.58	1,265
(3Z)-Hexenyl angelate	C_11_H_18_O_2_	0	0.19	0	1,277
Bornyl acetate	C_12_H_20_O_2_	0.27	0.57	0.29	1,285
2-undecanone	C_11_H_22_O	0.18	0	0	1,294
Perilla alcohol	C_10_H_16_O	0	0	0.32	1,295
Pinocarvyl acetate	C_12_H_18_O_2_	0	0.65	0	1,298
Myrtenyl acetate	C_12_H_18_O_2_	0.4	0	0	1,326
α-copaene	C_15_H_24_	0.27	0	0	1,376
Germacrene D	C_15_H_24_	0.55	0.38	0.75	1,481
Bicyclogermacrene	C_15_H_24_	0	0	0.27	1,500
Laciniata furanone H	C_15_H_22_O_3_	0.32	0	0	1,550
1-nor-bourbonanone	C_14_H_22_O	0.4	0	0	1,563
Davanone	C_15_H_24_O_2_	3.67	0	0	1,587
Ledol	C_15_H_26_O	0.16	0	0	1,602
α-cadinol	C_15_H_26_O	0.52	0	0	1,654
Dronabinol	C_21_H_30_O_2_	0	0	0.4	2,470

^a^ KI, kovats retention index.

The primary constituents of AH 1 essential oil are trans-thujone (56.73%) and cis-thujone (15.38%), with secondary components including camphor (6.75%), davanone (3.67%), and eucalyptol (2.29%). Conversely, the AH2 species found in Ifrane is distinguished by its high concentration of camphor (45.29%), with eucalyptol (11.68%), camphene (9.07%), and cis-thujone (8.96%) following as secondary components. In addition, the proportions of Santolina alcohol, Yomogi alcohol, and Artemisia alcohol are 4.08%, 3.08%, and 2.92% correspondingly. The AH 3 essential oil from Tata is mostly composed of camphor (37.57%), cis-thujone (27.38%), camphene (6.71%), trans-thujone (6.55%), and eucalyptol (5.23%). Figure 2B illustrates the mass spectra of the primary chemicals obtained from the examined *Artemisia* species. The investigated *Artemisia* species’ essential oils consist mostly of ketones, followed by non-aromatic alcohols and hydrocarbons. Additionally, the *Artemisia* species is characterized by different amounts of esters, aldehydes, and epoxides, which are additional groupings of chemical substances (Figure 3B).

### 3.3 Antimicrobial activity

The antimicrobial activity of *Artemisia* was evaluated against bacterial and fungal strains (Table 2). The obtained MIC results show that the studied *Artemisia* essential oils are more active against fungal strains compared to tested bacteria. Among the evaluated bacteria, *Enterobacter cloacae* strains were most sensitive to the different essential oils with a MIC of around 12.5 mg/mL. For fungi, AH3 and AH1 oils showed higher activity against various fungal strains compared to AH2 oil, with MIC values of 12.5 mg/mL. According to the MBC/MIC ratio, the studied *Artemisia* essential oils have bactericidal effects against bacteria and fungicidal effects against the tested fungi.

**TABLE 2:** MIC, MBC, and MFC of HE (mg/mL), antibiotics, and antifungals (µg/mL) against the microbial strains studied.

Microorganism		AH 1		AH 2		AH 3		Antibiotics^a^					Antifungals^b^
		MIC	MBC	MIC	MBC	MIC	MBC	Gentamycin	Amoxicillin–Clavulanate	Vancomycin	Trimethoprim-Sulfamethoxazole	Penicillin G	Terbinafine
*Bacteria*	*S. epidermidis*	100	100	25	50	12.5	25	2		>8	>4/76		
	*S. aureus BLACT*	50	50	25	25	12.5	12.5	<0.5		2	<10		
	*E. coli*	25	25	25	25	12.5	25	2	8/2		≤1/19		
	*E. cloacae*	12.5	12.5	12.5	25	12.5	12.5	>4	>8/2		>4/76		
	*K. pneumoniae*	25	25	25	25	12.5	12.5	≤1	≤2/2		≤1/19		
*Yeasts*	*C. albicans*	12.5	25	25	50	12.5	12.5						12.500
	*C. parapsilosis*	12.5	25	12.5	12.5	12.5	12.5						6.250
	*C. tropicalis*	12.5	25	12.5	12.5	12.5	12.5						12.500
	*C. dubliniensis*	12.5	25	12.5	12.5	12.5	12.5						3.125
*Molds*	*A. niger*	12.5	25	12.5	12.5	12.5	25						3.125

^a^ the minimum inhibitory concentration (MIC) of the antibiotics was measured using the BD, Phoenix™ identification and anti-biogram device.

^b^ the MIC, of terbinafine was calculated on a microplate.

### 3.4 Antioxidant activity

**FIGURE 4:**
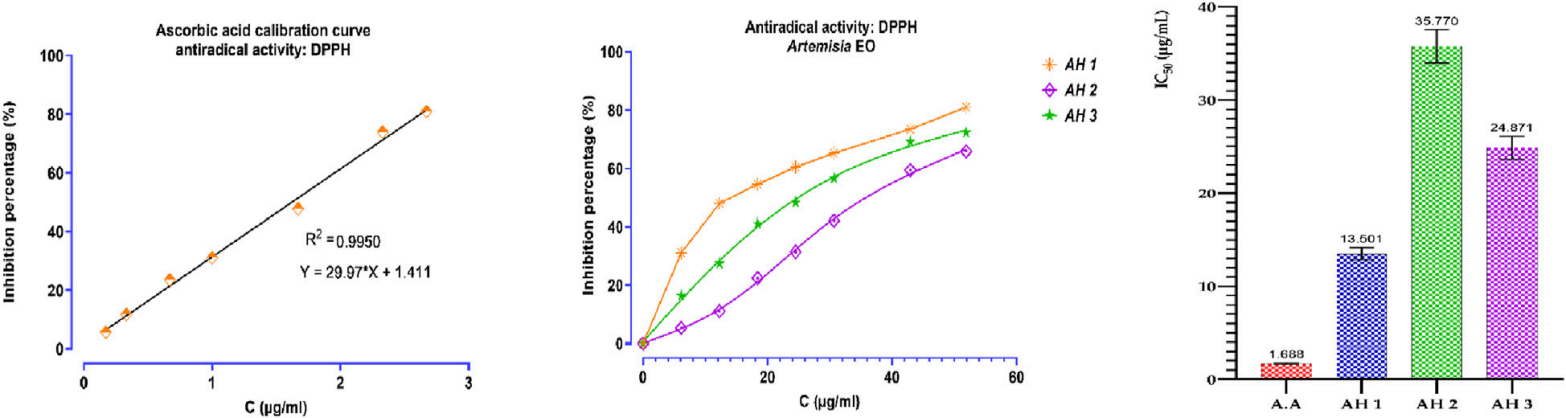
Antioxidant activity of EO from *Artemisia* species studied.

An assessment was conducted to determine the impact of several *Artemisia* essential oils on the DPPH radical. The three examined species of *Artemisia* shown the capacity to convert stable DPPH (2,2-diphenyl-1-picrylhydrazyl) into yellow diphenylpicrylhydrazine. This reduction capacity was determined by a decrease in absorbance induced by the antiradical substances present in the essential oils. As shown in the Figure below, the percentage inhibition of DPPH increases with the concentration of ascorbic acid and essential oils (Figure 4). Additionally, the antioxidant power of the different essential oils was evaluated by comparing IC_50_ values, determined in μg/mL, expressing the effective concentration of essential oil needed to trap and reduce 50% of DPPH moles dissolved in ethanol (Figure 4). The results obtained show that the tested essential oils exhibited remarkable antiradical activity, although lower than that of ascorbic acid. The ascorbic acid (A.A) showed an IC_50_ of 1.686 μg/mL. The best DPPH inhibition was recorded for AH1 oil with an IC_50_ of 13.501 μg/mL, followed by AH3 and AH2 oils (IC_50_ = 24.871 and IC_50_ = 35.770 μg/mL, respectively).

### 3.5 PASS, ADMET, and predictive methods to assess the effectiveness of potentially active chemicals extracted from *artemisia* EO

To produce therapeutic agents and support the efficacy of the chemical makeup of the plants under research as antioxidant and antimicrobial substances, it is imperative to investigate the physicochemical features of candidate compounds. An investigation of the components included in the essential oils was carried out to predict the compounds’ pharmacokinetic, physicochemical, and drug-like features.

The antibacterial and antioxidant activity of the major components isolated from the essential oils were predicted using the PASS prediction. Table 3 displays the results of the PASS and ADMET prediction trials. All compounds exhibited significant “Pa” values (Table 3) for various effects, such as antioxidant (0.161–0.399), antifungal (0.214–0.487), and antibacterial potential (0.191–0.463). Our predictions suggest that each significant compound has potent antibacterial, antifungal, and antioxidant properties.

**TABLE 3:** *In silico* study of the EO from the examined *Artemisia* species using PASS, ADMET, and predictive toxicity analysis (Pro-Tox II).

Prediction	Parameters	Camphene	Eucalyptol	Cis-thujone	Trans-thujone	Yomogi alcohol	Santolina alcohol	Camphor	Davanone
PASS Prediction (Pa/Pi)									
Antioxidant	Antioxidant	-	0.161/0.090	-	-	0.379/0.014	0.270/0.030	0.192/0.060	0.399/0.012
Antimicrobial	Antifungal	0.246/0.108	0.214/0.128	0.371/0.057	0.371/0.057	0.375/0.056	0.487/0.033	0.270/0.096	0.485/0.033
	Antibacterial	0.387/0.033	0.298/0.061	0.302/0.059	0.302/0.059	0.305/0.058	0.321/0.053	0.191/0.126	0.463/0.020
ADME Prediction									
Physiochemical Properties	TPSA (Å^2^)	0.00	9.23	17.07	17.07	20.23	20.23	17.07	26.30
	Molar Refractivity	45.22	47.12	45.90	45.90	50.18	50.44	45.64	72.48
Drug Likeness Prediction	Bioavailability Score	0.55	0.55	0.55	0.55	0.55	0.55	0.55	0.55
	Synthetic accessibility	3.50	3.65	2.79	2.79	2.88	3.15	3.22	3.96
Absorption Parameters Prediction	Water solubility	−4.34	−2.63	−2.995	−2.995	−2.341	−2.302	−2.598	−3.196
	Caco2 permeability	1.387	−2.63	1.375	1.375	1.49	1.501	1.112	1.367
	Intestinal absorption (human)	94.148	96.505	98.105	98.105	93.019	93.937	96.992	96.338
	Skin Permeability	−1.435	−2.437	−1.818	−1.818	−1.891	−1.958	−2.09	−2.276
	P-glycoprotein substrate	No							
	P-glycoprotein I inhibitor								
	P-glycoprotein II inhibitor								
Distribution Parameters Prediction	VDss (human)	0.547	0.491	0.345	0.345	0.117	0.118	0.266	0.192
	Fraction unbound (human)	0.547	0.553	0.396	0.396	0.517	0.514	0.459	0.389
	BBB permeability	0.787	0.368	0.71	0.71	0.575	0.584	0.619	0.55
	CNS permeability	−1.71	−2.972	−1.939	−1.939	−2.433	−2.453	−2.193	−2.67
Metabolism Parameters Prediction	CYP2D6 substrate	No							
	CYP3A4 substrate								
	CYP1A2 inhibitor								
	CYP2C19 inhibitor								
	CYP2C9 inhibitor								
	CYP2D6 inhibitor								
	CYP3A4 inhibitor								
Excretion	Total Clearance	0.049	1.009	0.135	0.135	0.471	0.521	0.109	1.512
	Renal OCT2 substrate	No							
Toxicity	AMES toxicity	No							
	hERG I inhibitors								
	Skin Sensitization								
	LD_50_ (mg/kg)	5,000	2,480	500	500	3,500	4,700	775	339
	Predicted Toxicity Class	5	5	4	4	5	5	4	4
	Hepatotoxicity	Inactive	Inactive	Inactive	Inactive	Inactive	Inactive	Inactive	Inactive
	Carcinogenicity					Active (0.68)	Active (0.65)		
	Immunotoxicity					Inactive	Inactive		
	Mutagenicity					Inactive	Inactive		
	Cytotoxicity					Inactive	Inactive		

**FIGURE 5:**
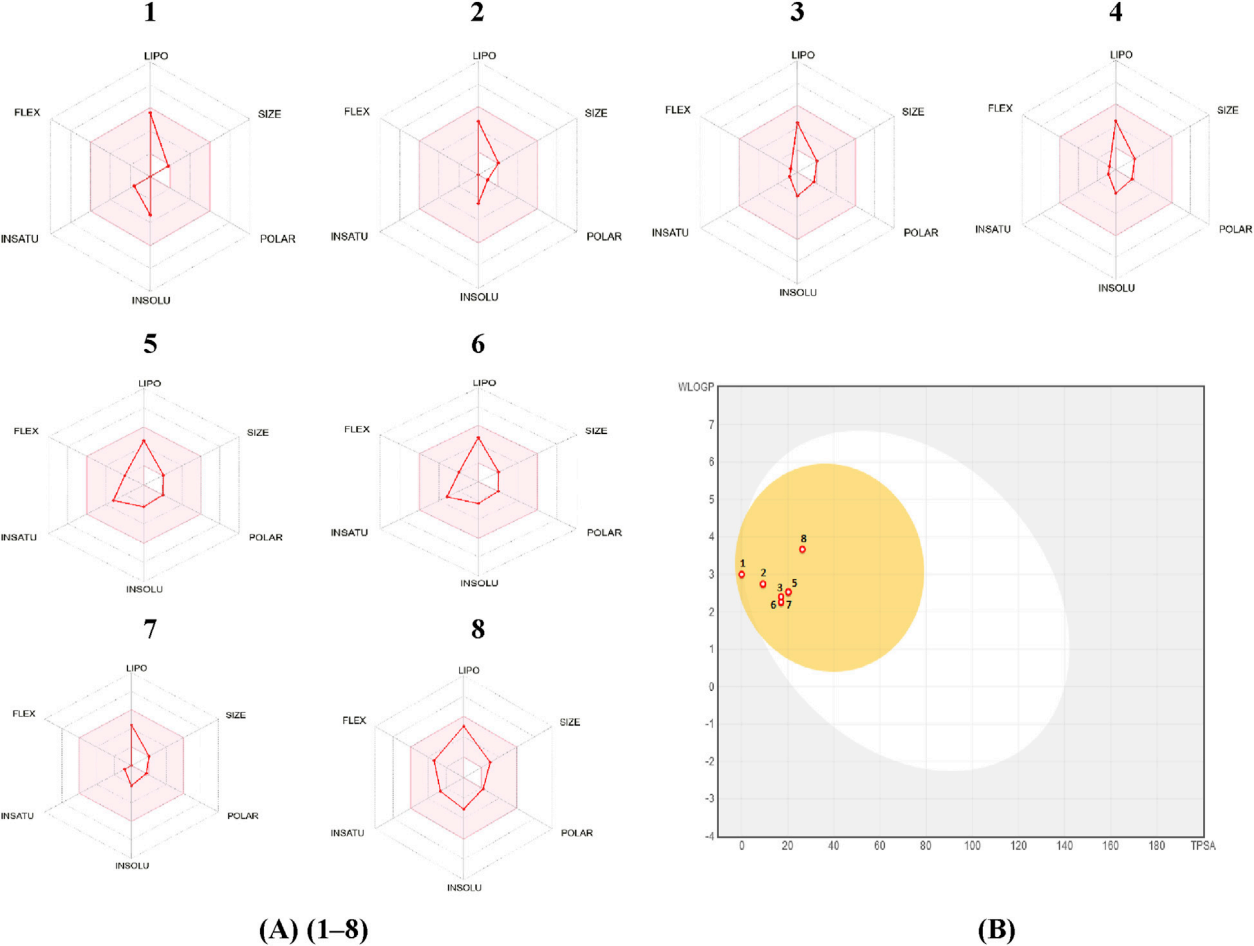
**(A)**: bioavailability radar, **(B)**: Predicted BOILED-Egg diagram of Camphene (1), Eucalyptol (2), cis-thujone (3), trans-thujone (4), yomogi alcohol (5), Santolina alcohol (6), Camphor (7), Davanone (8).

The web programs SwissADME and pkCSM are useful for understanding the physicochemical, pharmacokinetic, and structural similarities between substances and drugs. The Swiss-ADME web server was utilized to evaluate the radar plot of bioavailability, which depicts the pharmacokinetic, physicochemical properties, and drug similarity of compounds included in the analyzed *Artemisia* essential oils. The results are displayed in Figure 5.

The results of this study indicate that camphene, eucalyptol, cis-thujone, trans-thujone, yomogi alcohol, santolina alcohol, camphor, and davanone have a high ability to be absorbed by the gastrointestinal tract and passively diffuse through the blood-brain barrier (BBB).

Two approaches that may be employed to evaluate the drug-likeness of a molecule are molar refractivity and topological polar surface area (TPSA). Based on the prediction results, all the pertinent compounds found in the essential oils being studied, which had bioactivity scores of 0.55, met the drug-likeness criteria without any exceptions. The primary compounds being studied exhibit molar refractivity values that fall within the standard range of 40–130. Furthermore, the synthetic accessibility of these molecules (ranging from 2.79 to 3.96) indicates the presence of a unique synthesis pathway.

The lipophilicity indices of the compounds indicate that they possess a certain level of water solubility, albeit they are not completely soluble. This physicochemical characteristic is often beneficial for maintaining adequate absorption and bioavailability. The selected compounds exhibited favorable skin permeability values (log Kp) and elevated Caco-2 permeability values. Therefore, most medicines exhibit exceptional absorption in the gut (HIA >30%). P-glycoprotein, often known as P-gp, plays a vital role in the transportation and uptake of medications. None of the primary components of the investigated *Artemisia* essential oils exhibit inhibitory effects on P-gp I or P-gp II, nor do they act as substrates for P-gp.

Given that the log BB value is greater than 0.3 and the CNS score is higher than −3.0, it can be concluded that all of the isolated compounds from the essential oils being studied can easily cross the blood-brain barrier (BBB) and only a small amount can penetrate the central nervous system (CNS). The distribution volumes (logVDss) of these substances vary from 0.117 L/kg to 0.547 L/kg within tissues.

The process of medication elimination relies on the activity of cytochrome P450 (CYP) enzymes and molecular interactions. Therefore, it is improbable that there would be any harmful effects from drug interactions while orally consuming the essential oils being investigated. The total clearance (CLTOT) for renal organic cation transporters 2 (OCT2) and hepatic and renal substrates was estimated as the logarithm of the anticipated volume of distribution per unit of body weight, in order to predict the excretion pathway. The results demonstrated that each phytochemical component examined had a favorable overall clearance value and was capable of being excreted.

The phytochemical components of the studied *Artemisia* essential oils were examined to assess their potential toxicity in terms of AMES, hepatotoxicity, carcinogenicity, immunotoxicity, mutagenicity, cytotoxicity, hERG potassium channel inhibition, and skin sensitization. Except for Yomogi and Santolina alcohol compounds, which could have very little cancer-causing effects, the findings clearly show that there is no toxicity to cells, no influence on genetic mutations, and no harm to the liver.

### 3.6 Molecular docking study

In this investigation, the compounds identified using GC/MS were selected for *in silico* molecular docking studies due to the notably high *in vitro* biological activity of Artemisia essential oils. Molecular docking experiments were instrumental in predicting how these compounds interact with specific proteins linked to antioxidant, antifungal, and antibacterial functions. This interaction modeling allows us to infer how these compounds may inhibit or neutralize harmful microbial activities.

**FIGURE 6:**
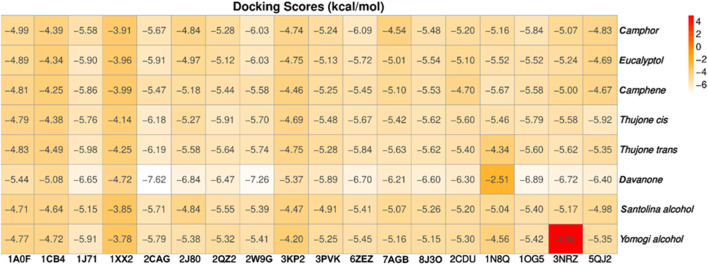
Heat map of binding affinities of antimicrobial and antioxidant proteins to selected ligands.

The study focused on analyzing key molecular interactions, such as van der Waals forces (VDW), hydrogen bonds, and C-H (carbon-hydrogen) bonds, which indicate how well a compound can bind to a target protein. Specifically, hydrogen bonds and van der Waals interactions contribute significantly to binding strength and stability, while C-H bonds and pi-sigma interactions further stabilize the compound-receptor complex. Binding strength is crucial, as it often correlates with a compound’s potential efficacy in disrupting or altering microbial functions. Figure 6 displays the docking energy binding scores among the target proteins’ binding sites for antifungal activities (5TZ1), antibacterial activities (1JZQ; 2VEG; 3RAE; 3SRW; 1KZN; 5OE3; and 4URN), and antioxidant activities (NADPH oxidase, 1N8Q; 1OG5; 3NRZ; and 5QJ2). This heat map visualizes binding affinities, with stronger binding affinities often suggesting higher potential bioactivity against these pathogens.

The compounds with the highest binding energies with the target proteins, indicating potential effectiveness, were davanone, eucalyptol, camphor, and the isomers of thujone. Davanone demonstrated substantial binding energy with protein 2cag for antibacterial activity (−7.62) and with protein 2w9g for antioxidant activity (−7.26), followed by eucalyptol, camphor, and the thujone isomers. These values reflect strong interactions, which imply that these compounds could effectively disrupt bacterial or antioxidant enzyme functions in pathogenic organisms.

The interactions between camphor and proteins 6ZEZ and 2W9G were characterized by hydrogen bonds, alkyl, and pi-alkyl bonds with specific amino acid residues such as ASP27, ALA7, PHE92, VAL606, GLY367, and ALA366 (Table 4). Hydrogen bonds are significant as they strengthen compound-protein attachment, enhancing camphor’s binding stability and potential activity against these proteins. Similarly, eucalyptol interacted with dihydrofolate reductase (2w9g) through pi-sigma, alkyl, and pi-alkyl linkages with residues like PHE92, LEU20, ALA7, and VAL31, adding to its stabilization and binding strength (Table 4).

**TABLE 4:** 2D and 3D interaction of ligands with target proteins.

Ligands	Target proteins	2D	3D
Camphor	6ZEZ	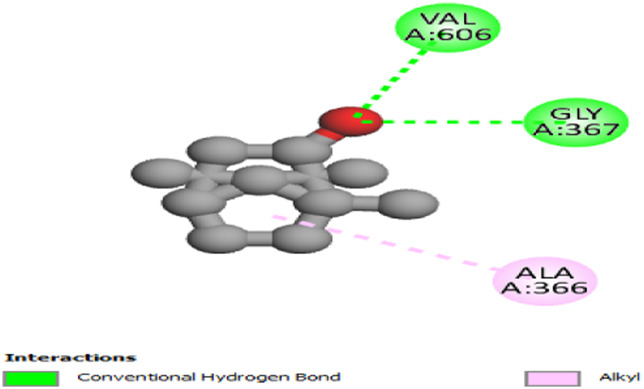	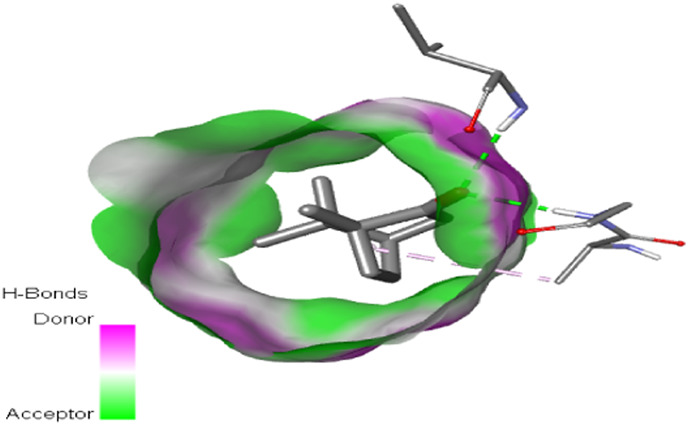
	2W9G	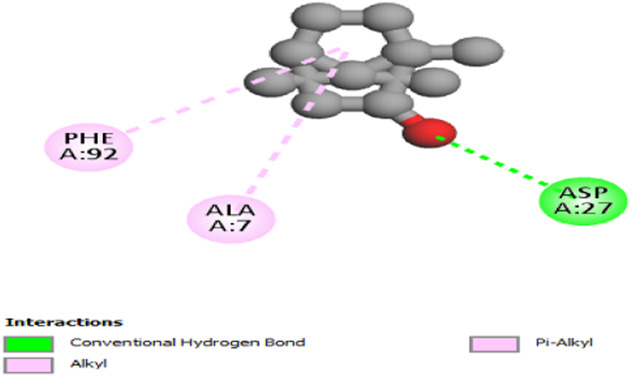	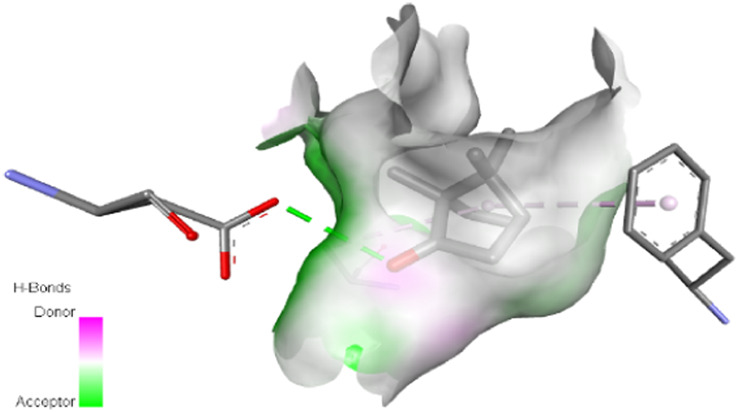
Eucalyptol	2W9G	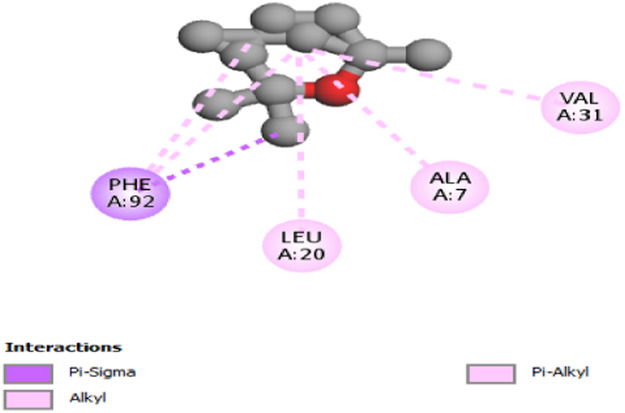	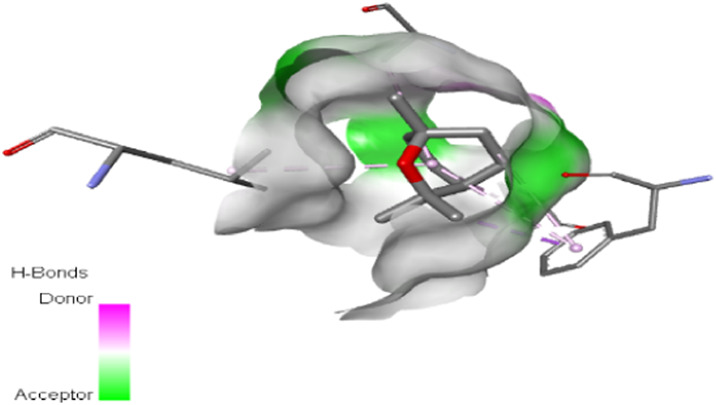
cis-thujone	2CAG	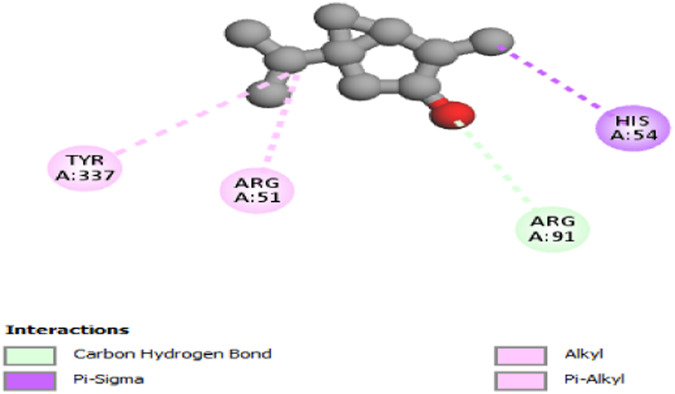	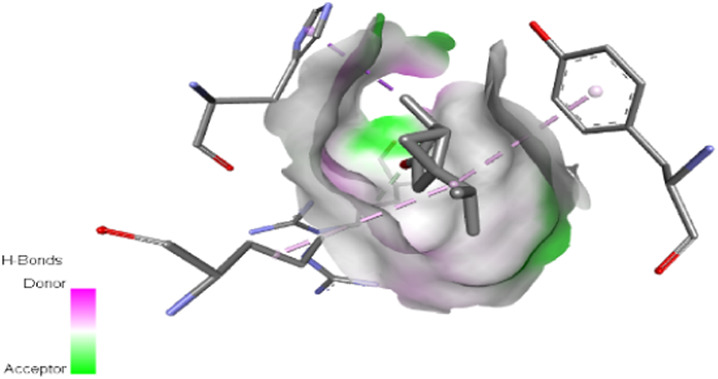
trans-thujone	2CAG	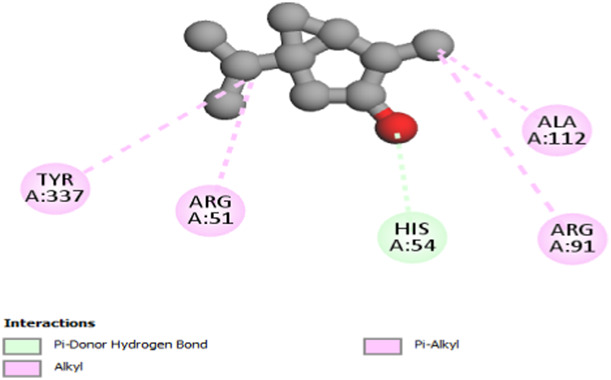	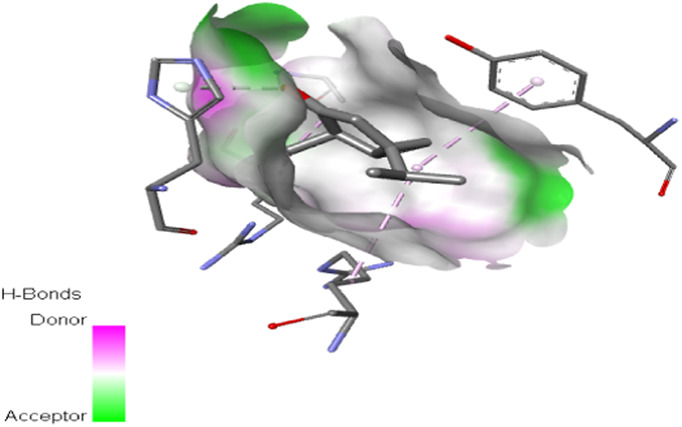
Davanone	2CAG	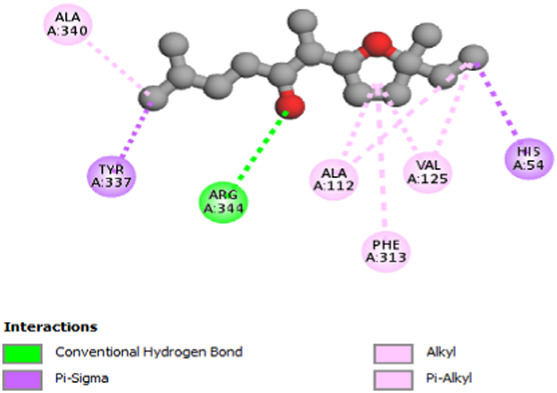	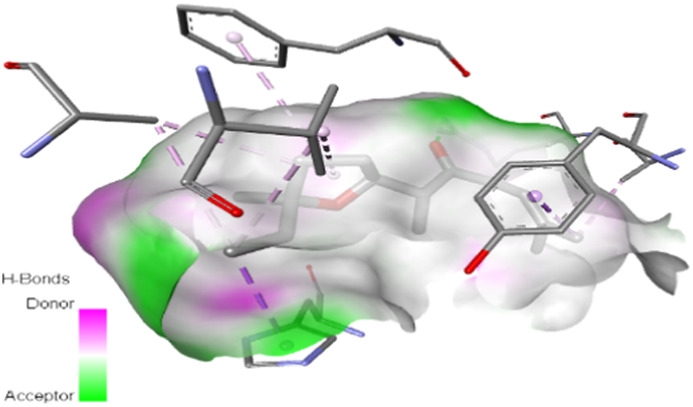
	2W9G	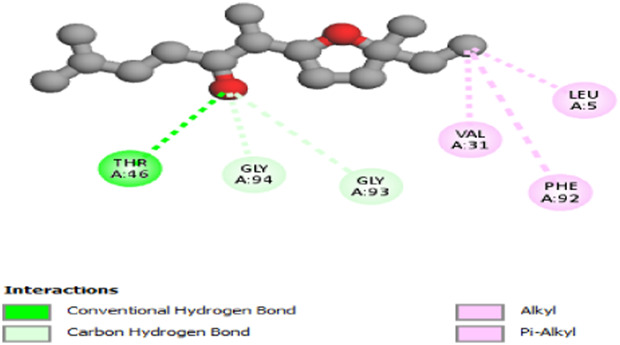	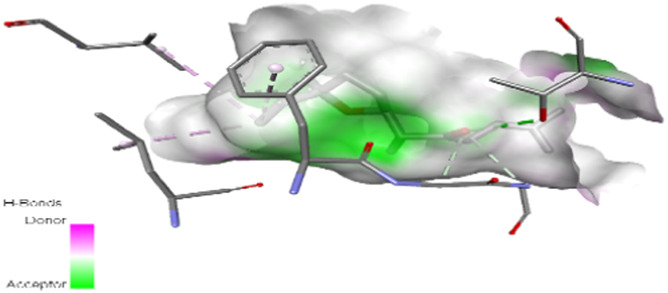

The thujone isomers (cis-thujone and trans-thujone) exhibited diverse interactions with protein 2cag. Cis-thujone interacted through carbon-hydrogen bonds, alkyl, and pi-alkyl bonds with residues ARG91, HIS54, TYR337, and ARG51, while trans-thujone’s interactions involved alkyl and pi-alkyl bonds with TYR337, ARG51, ARG91, and ALA112, as well as hydrogen bonds with HIS54 (Table 4). These binding differences indicate unique modes of interaction that could translate into distinct biological effects, depending on each isomer’s bonding characteristics.

Moreover, davanone’s interaction with catalase compound II (2cag) involved alkyl, pi-alkyl bonds, hydrogen bonds, and pi-sigma interactions with residues like ARG344, HIS54, TYR337, ALA340, ALA112, VAL125, and PHE313, while interactions with dihydrofolate reductase (2w9g) involved hydrogen bonds, C-H bonds, alkyl, and pi-alkyl linkages with THR46, GLY93, GLY94, VAL31, LEU5, and PHE92 (Table 4). These various binding types suggest that davanone can adapt flexibly across different protein targets, making it a versatile candidate in antimicrobial and antioxidant applications.

### 3.7 Molecular dynamic study

The process of molecular docking was employed to discover the protein-ligand complexes that showed the most potential. These complexes were subsequently selected for molecular dynamics (MD) simulations. The molecular docking results strongly correlate with PASS’s predictions of biological activity. The simulation results were analyzed using features such as the radius of gyration (Rg), root mean square fluctuation (RMSF), root mean square deviation (RMSD), and hydrogen bonding. Out of all the essential oils that were analyzed, davanone had the highest affinity for the target proteins. Hence, for a duration of 100 nanoseconds, the complexes 2CAG + davanone and 2W9G + davanone were chosen for molecular dynamics simulations.

#### 3.7.1 Structural dynamics of 2CAG

We conducted a thorough examination of the composition of *Proteus mirabilis* (PMC) NADPH-dependent catalase, specifically focusing on the protein 2cag. The dynamic changes in the structure of the 2CAG + davanone complex demonstrated a well-balanced simulation, as seen in Table 5A. During the 100 ns simulation, the root mean square deviation of protein 2CAG exhibited little variations, remaining close to the thermal average. In addition, the root mean square deviation (RMSD) values of the heavy atoms in davanone exhibited its stability relative to the protein. These values were significantly lower than those of the protein, suggesting that davanone remained in close proximity to its initial binding site. The interaction between davanone and protein 2CAG is significant, as indicated by the root mean square fluctuations detected during a 100 ns period, which are below 3 Å. Davanone does not seem to have a substantial impact on the structure of proteins. In addition, research of hydrogen bonding showed that davanone formed an average of 1.99 hydrogen bonds and 0.14 pairs within a 0.35 nm radius to the active pocket of 2CAG. Throughout the simulation, the radius of gyration values remained consistent, ranging from 1.85 to 2.2 Å. This indicates that the davanone compactness remained stable and demonstrates the appropriate flexibility of protein 2CAG after interacting with davanone. Based on our research, davanone demonstrates a strong affinity for protein 2CAG, without altering the protein’s structure, and maintains extended interaction with the protein’s binding sites.

**TABLE 5:** Structural dynamics of the 2CAG (A) and 2w9g (B) protein.

	RMSD	RMSF	Total number of intramolecular hydrogen bonds	Radius of gyration
A	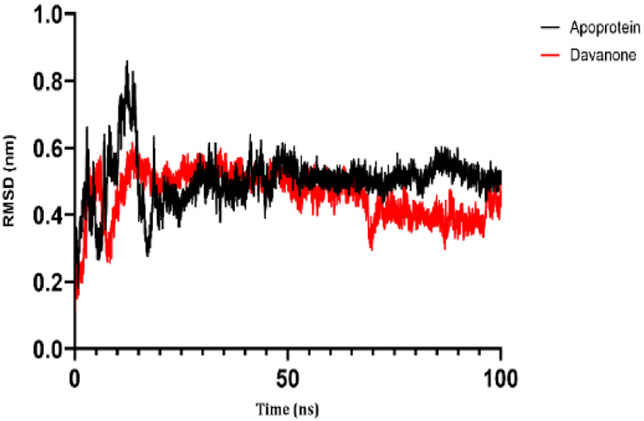	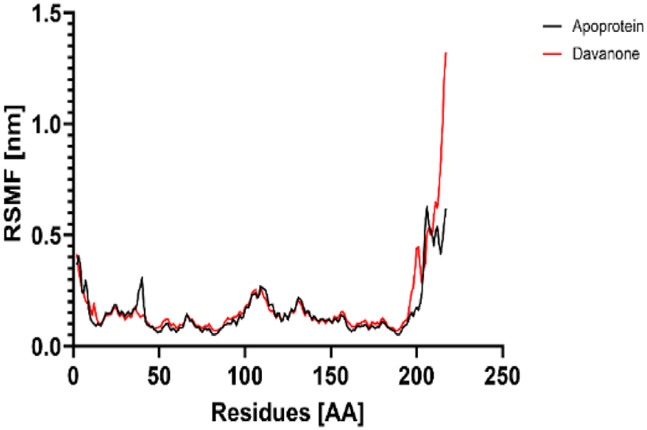	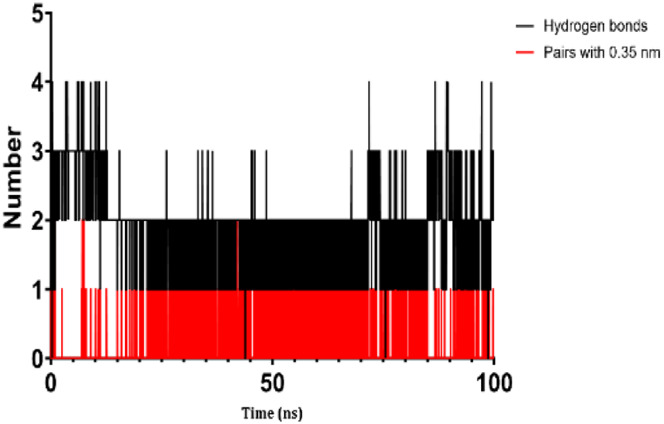	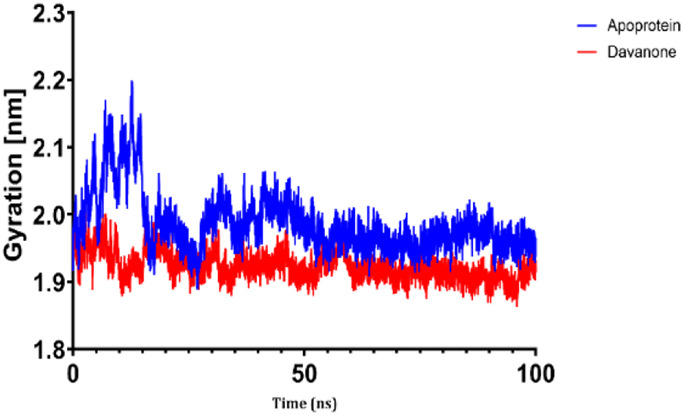
B	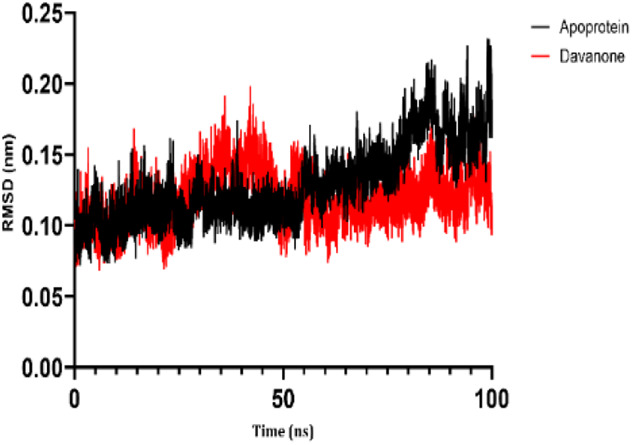	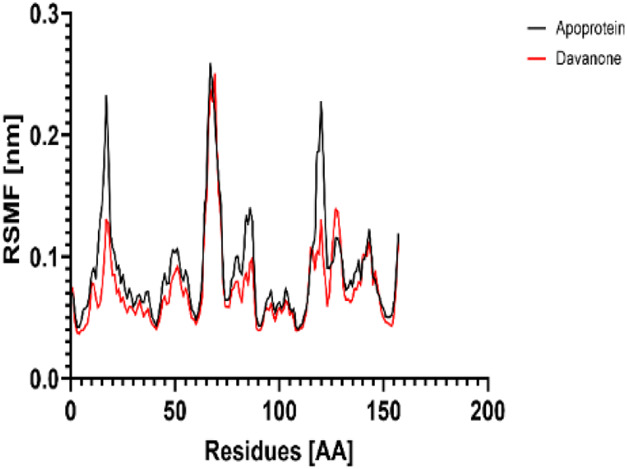	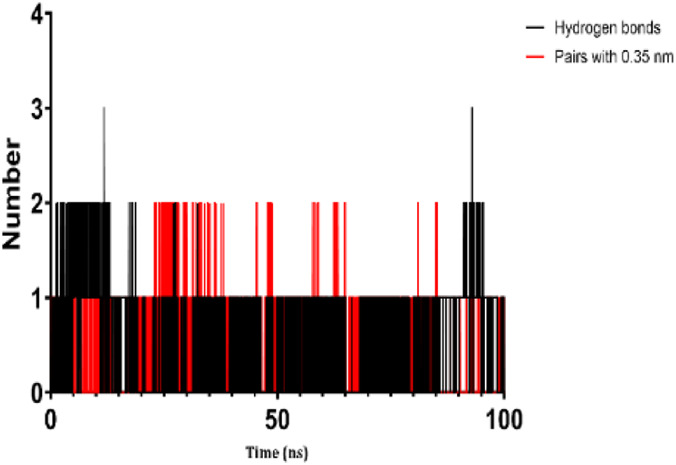	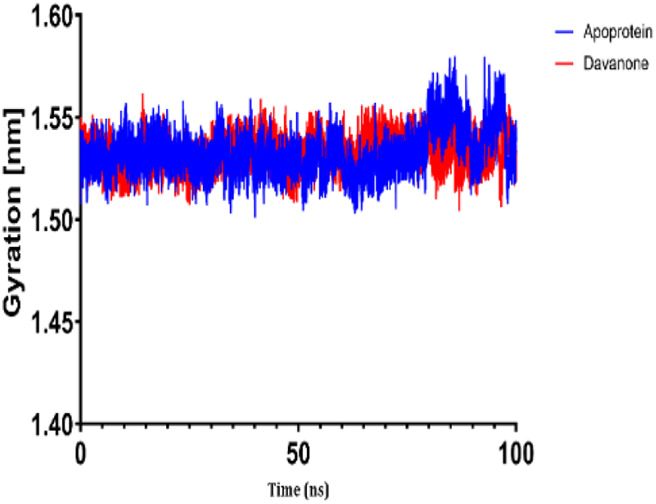

#### 3.7.2 Structural dynamics of 2W9G

Our findings show that the 2W9G + davanone complex was simulated in a balanced manner, with changes in the protein 2w9g’s root mean square deviation remaining quite near to the thermal average throughout the 100 ns simulation (Table 5B). Furthermore, davanone’s heavy atoms’ RMSD values show how stable it is in comparison to the protein; these values are substantially lower than the protein’s, indicating that davanone stayed securely anchored at its original binding site. Without appreciably changing protein structure, the root mean square changes over a 100 ns period stay below 3 Å, indicating a robust interaction between davanone and protein 2W9G. Furthermore, hydrogen bond analysis shows that davanone forms an average of 0.51 hydrogen bonds and 0.21 pairs within a radius of 0.35 nm when it binds closely to the active pocket of 2W9G. Until the simulation’s conclusion, the radius of gyration values fluctuated within a small range of 1.5–1.58 Å, showing that protein 2w9g was rather flexible following its interaction with davanone. These findings demonstrate a robust association that davanone maintains with the binding pockets of 2W9G and underscore a strong binding between davanone and dihydrofolate reductase, without dramatically altering protein shape.

The results obtained from molecular docking and *in vitro* experiments are supported by molecular dynamics simulations. The ligands being studied have high inhibitory potency, establishing stable connections with certain amino acids in the target proteins. The interactions between these entities result in the formation of complexes that remain stable during the whole 100 ns simulation, save for very small oscillations. Therefore, the utilization of both molecular docking and molecular dynamics has successfully verified the stability of the complexes created by the primary bioactive chemicals and their protein targets, thereby strengthening the significance of the discovered biological outcomes. This computational technique has supported the *in vitro* findings, confirming the inhibitory capability of the examined ligands and the structural basis of their interactions with the identified protein receptors.

## 4 Discussion

Due to their medicinal properties and low toxicity, essential oils have experienced a significant increase in demand throughout time as they are utilized to treat many serious conditions. The aim of our study was to examine the antibacterial and antioxidant properties of the essential oils (EO) derived from *Artemisia* species. The plant species known as *A. herba-alba* Asso and *A. huguetii* Caball. is a plant species that is native to the Mediterranean area, namely Morocco.

The three *Artemisia* species had different yields from the EO depending on where they were grown. The yield percentages of the EO of the species *A. herba-alba* Asso from Boulemane (AH 1) and Ifrane (AH 2) reached 1.54% and 2.09%, respectively, while the yield of the EO from *A. huguetii* Caball. fom Tava (AH 3) recorded a higher percentage of 2.78%. The percentages observed in this study were greater than those reported in earlier investigations on *Artemisia* species. Specifically, we observed mean yields of 0.86% and 1.19% for *A. herba-alba* plant is found in the Azzemour area (Bakha et al., 2018) and the Mouzzer Marmoucha region (Btisam et al., 2016). Nevertheless, our findings closely align with the results reported by Majdouli (Majdouli and Elhazzouzi, 2015) on the *A.* huguetii Caball. was collected in the Tata area and had a rate of 4.28%. However, the differences in the amount of EO produced may be attributed to a wide range of intricate and interconnected elements, which have been extensively studied and improved upon in recent years. Factors such as drying conditions, harvesting season, geographical location, fertilizer, soil pH, as well as intrinsic plant factors like chemotype, plant part employed, and genotype, have all been demonstrated to have a significant impact (Louhaichi et al., 2015; Elwardani et al., 2024). Furthermore, the choice of extraction process is also a crucial factor (Chebbac et al., 2023). Moreover, recent advancements in optimizing extraction techniques and exploring nanoencapsulation have proven promising in enhancing the efficacy and stability of essential oils, thereby maximizing their bioactive potential (Wani et al., 2024).

The species under examination predominantly consist of oxygenated monoterpenes, as determined by GC/MS analysis of the EO. In contrast, there were low levels of sesquiterpenes and diterpenes, along with lower amounts of hydrocarbon monoterpenes. Prior research has demonstrated that the majority of white *Artemisia* essential oils consist mostly of hydrocarbon and oxygenated monoterpenes, with sesquiterpenes being a close second in composition. Our findings are consistent with this research. Similar quantities have been reported by studies in several populations of *A. herba-alba* in Morocco by Benjilali (1982); and by Amor et al. (2019); in Spain by Salido et al. (2004); in Tunisia by Bellili et al. (2016); as well as in other species of *Artemisia* found worldwide, including *A. absinthium* via the work of Vieira et al. (2017), in Brazil; *A. sieberi* by Mohammadhosseini et al. (2016), in Iran; and *A. nilagirica* by Sandip et al. (2014), in India.

Furthermore, according to GC-MS analysis, we observed that the studied essential oils are rich in active substances. For example, *A. herba-alba* asso from Boulemane is rich in trans-thujone (56.73%), cis-thujone (15.38%), Camphor (6.75%), davanone (3.67%), and eucalyptol (2.29%). In contrast, in the species *A. herba-alba* Asso from Ifrane, camphor (45.29%), eucalyptol (11.68%), camphene (9.07%), cis-thujone (8.96%), Santolina alcohol (4.08%), Yomogi alcohol (3.08%), and *Artemisia* alcohol (2.92%) are found. Similarly, the EO of *A. huguetii* Caball. fom Tata is characterized by camphor (37.57%), cis-thujone (27.38%), camphene (6.71%), trans-thujone (6.55%), and eucalyptol (5.23%). It is important to note that most of these components are present in EOs extracted from *Artemisia* species collected in the Mediterranean region (*A. arborescens*, *A. caerulescens* subsp., and *A. annua*) (Ornano et al., 2013; Zhigzhitzhapova et al., 2020). These components are also present in EOs from *Artemisia* species in other countries such as Korea, Croatia, China, and Turkey (Kordali et al., 2005; Hong et al., 2023; Ma et al., 2023; Politeo et al., 2023).

Thujone (both trans- and cis-), camphor, eucalyptol, camphene, and davanone are the main components of the essential oils found in *Artemisia*. These ingredients have been well acknowledged (Bora and Sharma, 2011). There is a limited amount of literature available on the investigation of essential oils derived from Mediterranean plants, as shown by a small number of research studies (Ornano et al., 2013). It is important to mention that there have been reports of variations in the concentration of essential oils among subspecies due to the presence of various chemotypes within species. Interestingly, several chemicals in Moroccan *A. herba-alba* var huguetti have been identified by GC-MS, suggesting that this species may have its native origins in Morocco (Sanae et al., 2020). According to this, it appears that the *Artemisia* species being studied show chemical polymorphism, with different chemotypes linked to both inherent and external factors.

In terms of antibacterial activity, our findings align with previous studies (Chebbac et al., 2021; Ouguirti et al., 2021), which have demonstrated that *Artemisia* essential oils possess antimicrobial properties. The EO AH 3 demonstrated a higher level of antibacterial efficacy in comparison to AH 1 and AH 2. The observed antimicrobial activity in the investigated essential oils is mostly attributed to their significant concentration of oxygenated monoterpenes and hydrocarbon monoterpenes, which are well-known for their antibacterial properties (Habibi et al., 2013; Ouguirti et al., 2021). Therefore, the presence of davanone, a chemical compound absent in other species, is responsible for the potent antibacterial properties of EO AH 3, surpassing those of other essential oils being investigated.

The results align with the findings of Habibi et al. (2013), who provided evidence of the potent antibacterial properties of *A. herba-alba* essential oils have antibacterial activity against many types of bacteria, including E*scherichia coli, Klebsiella pneumoniae,* and *Staphylococcus aureus*. Additionally, our research is consistent with other investigations, such as the one conducted by Radulović et al. (2013), which discovered that essential oils containing a significant amount of ketone molecules, similar to the ones utilized in our investigation, exhibit antibacterial effects against dangerous microorganisms. Research has demonstrated that, as compared to Gram-positive cocci, Gram-negative bacteria are more vulnerable to the impacts of essential oils (Drioiche et al., 2022).

The antibacterial powers of essential oils are attributed to their lipophilic character, which enables them to effectively penetrate bacterial cells. It has been shown that the ketonic chemical compounds included in the essential oils of the investigated *Artemisia* species mostly gather in the cytoplasmic membranes. This leads to a change in the permeability of these membranes, resulting in the quick demise of the microorganisms (Amrati et al., 2021). Prior research has shown that essential oils have a greater antibacterial effect compared to the individual chemicals being studied. This suggests that the phytochemical components found in the essential oils, such as cis and trans-thujone and davanone, may work together in a synergistic manner rather than acting independently (Bilia et al., 2014; Yu et al., 2020).

The high concentration of thujone and davanone in the essential oil, which is frequently responsible for antifungal activity, along with minor compounds that may also significantly contribute to this activity, can be used to explain the essential oil of *A. huguetii* Caball., which was harvested from the Tata region and had potent antifungal activity when compared to other studied essential oils (Obistioiu et al., 2014).

Based on specific study, the antibacterial effectiveness of essential oils may be higher than that of their main components when analyzed separately (Bakkali et al., 2008). The activation of essential oils often occurs due to the synergistic effect of minor components, which is worth mentioning. Moreover, Agour’s study (Agour et al., 2021) has demonstrated a direct correlation between the existence of oxygenated terpenes and the effectiveness against bacteria, which aligns with our own findings regarding the oxygenated monoterpenes found in the essential oils we analyzed.

The IC_50_ values of the antioxidant activities described in this investigation align with the findings published by Benamar-Aissa et al. (2023). The essential oil of *Artemisia* species possesses significant antioxidant properties. The *A. herba-alba* Asso plant, collected in the Boulemane region, contains a high concentration of bioactive compounds such as thujone, camphor, and davanone. These chemicals have demonstrated significant antioxidant properties, equivalent to ascorbic acid, as documented in previous studies (Mrabti et al., 2017; Diass et al., 2023). Prior research has demonstrated that the antioxidant properties of essential oils are often associated with their primary constituents (Tepe et al., 2005; Baser and Buchbauer, 2009). Bakkali and Chebbac (Bakkali et al., 2008; Chebbac et al., 2022) have showed that the collective effect of small molecules can impact the antioxidant strength of essential oils. Essential oils rich in oxygenated mono-terpenes often have a greater capacity to eliminate radicals compared to those containing hydrocarbon monoterpenes (Woerdenbag et al., 1993). The results on antioxidants are consistent with prior research, which has shown that the *Artemisia* genus has significant antioxidant activity in many biological assays, such as the DPPH test, β-carotene bleaching, and total antioxidant capacity (Chebbac et al., 2021). The investigated *Artemisia* species include antioxidants that can effectively eradicate free radicals inside the human body, potentially preventing many chronic ailments such as cancer, cardiovascular disease, and premature aging. Therefore, the antioxidants included in *Artemisia* have the ability to safeguard cells from oxidative damage, perhaps leading to positive effects on health.

Molecular docking studies are frequently employed to forecast the interaction between a ligand and a protein, offering valuable insights into the antibacterial efficacy of natural sources. Furthermore, they offer insights into the interactions and probable mechanisms of action at different protein binding sites (Khan et al., 2019). Docking analysis was used to examine the essential oils of the *Artemisia* species in order to identify their primary components and understand the underlying processes responsible for their antibacterial and antioxidant properties. The study included the following receptor proteins: 1a0f, 1cb4, 1j71, 1xx2, 2cag, 2j80, 2qz2, 2w9g, 3kp2, 3pvk, 6zez, 7agb, 8j3o, 2CDU, 1N8Q, 1OG5, 3NRZ, and 5QJ2. The basic chemicals combine to create bonds that improve the stability of the ligand-protein complex, hence enhancing the strength of their interaction. Specific interactions, such as strategically positioned hydrogen bonds and adequate hydrophobic regions, enable an increased affinity between the ligand and the protein. This can have a positive impact on the compound’s ability to influence the biological processes under investigation. Compounds that have a small molecular weight, low affinity for lipids, and limited capacity to form hydrogen bonds may demonstrate improved absorption, permeability (Duffy et al., 2015), and bioavailability (Lipinski et al., 2001; Daina et al., 2014). Considering that the majority of the primary compounds under investigation meet Lipinski’s criteria, they possess the capacity to function as valuable reservoirs of antioxidants and antimicrobials.

The molecular docking investigations have revealed significant interactions between camphor, eucalyptol, davanone, and thujone (cis and trans) with several protein targets associated with antioxidant and antibacterial activities. Davanone, camphor, and eucalyptol have shown a significant attraction to dihydrofolate reductase, which is a crucial enzyme in DNA synthesis necessary for the development of bacterial and human cells. Thus, it is possible that microbial growth and cytotoxicity both have this enzyme as a common target. Moreover, due to their potent antioxidant properties, *Artemisia* species that contain significant levels of these compounds have the potential to inhibit protein oxidation and protect cells from damage. Davanone and thujone (cis and trans) have shown a significant affinity for catalase (2cag), suggesting their potential involvement in cellular protection. This affinity may be attributed to their ability to eliminate free radicals and activate signaling pathways, leading to the overexpression of genes and increased activity of antioxidant enzymes, specifically catalase. Moreover, davanone has shown significant affinity for aspartic proteinase, sensor kinase cita, endo-1,4-beta-xylanase I, kelch-like ECH-associated protein 1, candidapepsin, formate dehydrogenase, NADPH oxidase, cytochrome P450 2C9, xanthine dehydrogenase, and myeloperoxidase. These interactions suggest that davanone may hinder the function of these proteins, disrupting crucial bacterial survival pathways and enhancing the activity of antioxidant enzymes. Studies conducted by Houti et al. (2023), Singh et al. (2021), Brodin et al. (2007) and Vlatka et al. (2004), have shown that davanone, eucalyptol, camphor, and thujone (cis and trans) exhibit activity against various bacteria and fungi. The exact mechanism of action is uncertain, although it is believed to include either the inhibition of crucial enzymes or the bursting of cell membranes. Davanone has demonstrated its capacity to eliminate free radicals, so potentially protecting cells from the detrimental consequences of oxidative stress (Mohammed, 2022). This might be beneficial for several conditions, such as age-related ailments and neurological diseases.

The results of molecular docking studies and *in vitro* tests are supported by the results of molecular dynamics of complexes such as 2CAG + davanone and 2W9G + davanone. The idea that these substances are among the most potent inhibitors is supported by this data, which demonstrates that davanone forms consistent interactions with target proteins. This observation highlights how crucial it is for dynamically stable complexes to form during 100 ns simulations to potentially develop new antioxidant and antimicrobial therapeutic agents, especially from the essential oil of *A. herba-alba* Asso harvested in the Boulemane region. This study demonstrates the significant effects of essential oils on oxidative stress and bacterial cells due to their hydrophobicity, which makes bacterial cells more permeable and allows leakage of cytoplasmic components. Additionally, essential oils increase the activity of antioxidant enzymes by promoting the overexpression of their genes, especially those of catalase, NADPH oxidase, cytochrome P450 2C9, xanthine dehydrogenase, and myeloperoxidase.

*Artemisia* species are frequently used in traditional medicine because their essential oils have demonstrated antibacterial and antioxidant properties. They are well-known for their ability to produce bioactive chemical compounds, antifungal proteins, and peptides that combat several illnesses, including fungus (Farzaneh et al., 2006; Bisht et al., 2021). Chemicals that have notable antibacterial effects include 1,8-cineole, davanone acetate, camphor, camphene, and thujone (Abad et al., 2012; Chehregani et al., 2013). The biological activity shown in our data can be attributed to these chemicals, however it is likely that this activity is due to synergistic interactions with other components found in the investigated oil. Therefore, it is challenging to establish a direct relationship between the activity of a complex combination and a single element. This is because even little quantities of some compounds can significantly affect the reported antimicrobial activity (Vaou et al., 2021). Furthermore, while evaluating the biological activity of essential oils, it is vital to consider the potential synergistic and antagonistic effects of many compounds, since these may affect the ability to suppress bacteria. Essential oils are employed in dental care, with Listerine being the most widespread illustration. Listerine was developed in the 19th century as a potent antiseptic for surgical procedures (Fine, 2010). However, these recommendations may lack a strong scientific basis as they often come from empirical approaches. The results indicate that the compounds included in the examined *Artemisia* essential oils, such as davanone, eucalyptol, camphor, and thujone (cis and trans), may have a similar target or facilitate entrance to the location where they take effect. This strategy shows great potential for creating novel antioxidant and antibacterial therapies using these chemical molecules.

## 5 Conclusion

The results of our thorough examination into the chemical compositions of the essential oils of *A. herba-alba* Asso and *A. huguetii* Caball. fom Morocco revealed the significant presence of thujone isomers, camphene, eucalyptol, camphor, and davanone. The essential oils exhibited potent antibacterial properties against a range of microorganisms, underscoring their ability to kill bacteria and fungi. Additionally, our analysis showed that these essential oils had strong antioxidant activity and a remarkable ability to neutralize free radicals. The positive characteristics of ADMET indicate significant potential for therapeutic uses in the future. These findings are further supported by molecular docking experiments, which validated the biologically meaningful interactions with target proteins, thereby clarifying their likely mechanism of action.

The significant antibacterial and antioxidant properties identified in the essential oils of *A. herba-alba* Asso from the Boulemane and Ifrane areas, together with *A. huguetii* Caball. fom the Tata region, highlight their potential uses outside laboratory environments. These oils, abundant in thujone, camphene, eucalyptol, camphor, and davanone, have significant promise for the formulation of natural medicinal solutions intended for infection management and oxidative stress reduction. Such applications might meet the increasing need for alternative medicines, particularly in light of growing antibiotic resistance. Furthermore, these essential oils may function as natural components in cosmetic compositions with antibacterial and antioxidant properties, perhaps resulting in innovative skincare or preservation solutions with less synthetic additives.

Additional investigation into the formulation and safety profiles of these compounds may facilitate the utilization of these essential oils as active ingredients in innovative therapeutic or cosmetic products, translating scientific knowledge into practical applications that enhance public health and consumer markets.

## Data Availability

The original contributions presented in the study are included in the article/supplementary material, further inquiries can be directed to the corresponding authors.
